# From Laboratory to Field: Unsupervised Domain Adaptation for Plant Disease Recognition in the Wild

**DOI:** 10.34133/plantphenomics.0038

**Published:** 2023-03-28

**Authors:** Xinlu Wu, Xijian Fan, Peng Luo, Sruti Das Choudhury, Tardi Tjahjadi, Chunhua Hu

**Affiliations:** ^1^ College of Information Science and Technology, Nanjing Forestry University, Nanjing 210037, China.; ^2^ Institute of Forest Resource Information Techniques, Chinese Academy of Forestry, Beijing 100091, China.; ^3^ Key Laboratory of Forestry Remote Sensing and Information System, National Forestry and Grassland Administration, Beijing 100091, China.; ^4^ Department of Computer Science and Engineering, University of Nebraska-Lincoln, Lincoln, NE 68588, USA.; ^5^ School of Engineering, University of Warwick, Coventry CV4 7AL, UK.

## Abstract

Plant disease recognition is of vital importance to monitor plant development and predicting crop production. However, due to data degradation caused by different conditions of image acquisition, e.g., laboratory vs. field environment, machine learning-based recognition models generated within a specific dataset (source domain) tend to lose their validity when generalized to a novel dataset (target domain). To this end, domain adaptation methods can be leveraged for the recognition by learning invariant representations across domains. In this paper, we aim at addressing the issues of domain shift existing in plant disease recognition and propose a novel unsupervised domain adaptation method via uncertainty regularization, namely, Multi-Representation Subdomain Adaptation Network with Uncertainty Regularization for Cross-Species Plant Disease Classification (MSUN). Our simple but effective MSUN makes a breakthrough in plant disease recognition in the wild by using a large amount of unlabeled data and via nonadversarial training. Specifically, MSUN comprises multirepresentation, subdomain adaptation modules and auxiliary uncertainty regularization. The multirepresentation module enables MSUN to learn the overall structure of features and also focus on capturing more details by using the multiple representations of the source domain. This effectively alleviates the problem of large interdomain discrepancy. Subdomain adaptation is used to capture discriminative properties by addressing the issue of higher interclass similarity and lower intraclass variation. Finally, the auxiliary uncertainty regularization effectively suppresses the uncertainty problem due to domain transfer. MSUN was experimentally validated to achieve optimal results on the PlantDoc, Plant-Pathology, Corn-Leaf-Diseases, and Tomato-Leaf-Diseases datasets, with accuracies of 56.06%, 72.31%, 96.78%, and 50.58%, respectively, surpassing other state-of-the-art domain adaptation techniques considerably.

## Introduction

Plant diseases cause considerable financial loss to the global agricultural industry, especially in regions with harsh environments. Not only they pose a threat to global food security, they often result in disastrous consequences for smallholders who depend on healthy crops for their livelihoods. Thus, accurate recognition of different plant diseases is helpful in guiding farmers to take timely and appropriate steps to guarantee yield and quality and preventing loss in food production [1].

The traditional methods of disease recognition are divided into 2 main approaches: manual inspection by former or trained experts and image processing-based machine detection [2]. The manual inspection requires the experts to observe changes from the appearance of plant leaves, which is time-consuming and might lead to errors. Machine detection-based methods tend to preprocess the leaf images by employing traditional image processing strategies, e.g., image denoising, morphological analysis, and histogram equalization. Hand-crafted feature extraction is then applied to capture low-level information of target such as color, shape, and texture [3]. However, traditional machine detection-based methods simply extract shallow spatial information, without considering excavating the intrinsic relationship within the same disease categories. In addition, such techniques need prerequisite relevant knowledge for more useful representations, which lacks the generalization ability for different environments.

With the development of machine learning techniques, data-driven-based computer vision methods have increasingly been exploited to identify various plant diseases, which enable the effective classification of plant diseases with adequately labeled data in the set of training data [4–6]. In recent years, various deep convolutional neural network (CNN) structures have gained promising results in the classification of plant diseases [7–11]. These structures tend to contain thousands or even millions of parameters, requiring massive annotated samples to train the discriminative representation of a disease in a plant image. However, one of the problems is that it is nontrivial to collect high-quality annotated data [12,13], especially from the field environment. This is because some plant diseases are relatively rare or subtle and thus not easily observed. In addition, plant disease samples in the wild tend to suffer from their complex background, occlusions, and varied sizes and shapes, which introduce additional challenges to the annotating process by experts. Accordingly, annotating sufficient image samples for training a CNN classification model is time-consuming and labor-intensive. Another major problem in developing a CNN-based plant disease classification method is that CNNs are weak at generalizing the learned model or knowledge with a specific dataset (source dataset) to new datasets or environments (target dataset). For example, a classification network trained on a dataset collected under laboratory environment would show an apparent performance degradation when applied to a new dataset collected from the field. This is because of the distribution divergence between source and target datasets that is posed by different image acquisition circumstances, i.e., illumination, acquisition sensors, locations, etc. Such distribution discrepancy is usually referred to as the domain shift or distributional shift.

To address the aforementioned 2 problems, i.e., difficulty in acquiring good annotated images and domain/dataset shift, we apply one of the transfer learning techniques, unsupervised domain adaptation (UDA) [14–17], to the task of plant disease classification. UDA enables the transfer of the trained model using sufficient annotated samples from the source domain/dataset to a different but related target domain/dataset where only unannotated data are available. Concerning the task of plant disease classification, the images collected from the laboratory are usually of good quality, e.g., with a plain background, stable illumination, and uniform size, making them easy to be annotated. Thus, we can train a model for classifying plant disease by using the annotated images collected in the laboratory and employ UDA to transfer the learned meaningful and discriminative knowledge to the classification of plant disease images from the complex field environment. Compared with general plant disease classification methods without using UDA, annotation of field plant disease images in our case is not required, which largely reduces the time and labor involved. Moreover, the trained model is applicable to different unseen field environments for generating the specific model for each domain with only a set of unannotated data.

Although being widely studied in the community of computer vision owing to its tremendous advantages, research in domain adaptation on plant disease classification is rare. We only found 2 related studies [13,18]. Yan et al. [13] propose a cross-species plant disease recognition framework using UDA, where a mixed subdomain alignment method is employed to solve the multiclassification task of plant disease severity. Fuentes et al. [18] propose domain adaption for tomato disease classification, which can cope with changes of the environments. However, both of these 2 methods directly apply the existing UDA to classify plant diseases, thus failing to consider some issues exclusively existing in various plant diseases of cross species. These issues could negatively affect the performance of model transfer when using UDA for plant disease classification, which needs to be considered. These issues are as follows.

1. Large interdomain discrepancy

**Fig. 1.**
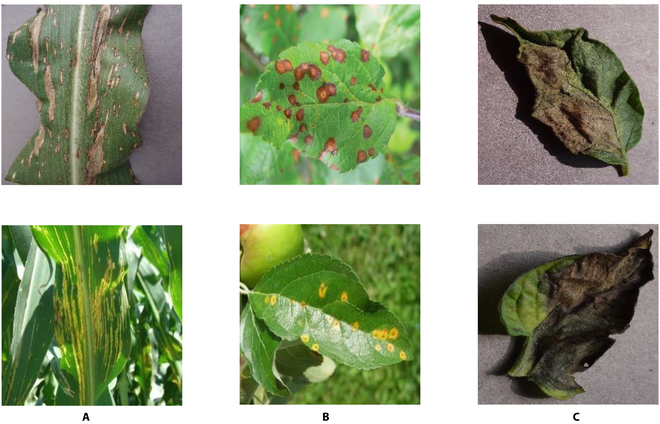
Issues existing in various cross-species plant diseases. The top and bottom images in column (A) are from the source domain (PVD) and the target domain (PlantDoc dataset). The images in column (B) are apple leaves with rust disease. In column (C), the top and bottom images are respectively potato leaf and tomato leaf with late blight disease.

As shown in Fig. 1A, the image collected under the laboratory environment is the source domain, containing only 1 plant leaf in an image with a single background, good illumination, and high-image resolution. In contrast, the image belonging to the target domain is collected from the field environment (referred to as “in the wild”), containing more than 1 leaf with a complex scene background, poor camera shooting angle, blurred image, and incomplete leaf display, thus introducing irrelevant information for disease classification and inducing the negative transfer.

2. Large intraclass discrepancy within a domain

Figure 1B shows plants of the same species suffering from varying states of the same disease and the disease manifestation occurring at varying sites due to diverse living environments and differences in the age of the plants. This intraclass discrepancy within a domain poses a challenge to the classifier.

3. Fuzzy boundary between classes in a domain

The same plant suffering from different diseases or the same disease on different plants may display similar manifestations (as shown in Fig.1C). This leads to the fact that clusters among different classes in the mapping space might be close to each other, and the global alignment thus fails to identify clear semantic structures confidently.

The above 3 issues can be characterized as uncertainty in classifying various diseases across species where the intrinsic semantics of images are difficult to maintain during subdomain adaptation. To better resolve this problem, more representations are considered when aligning the feature distribution of the datasets to offer a better insight on the inherent global structure in the source domain. Furthermore, to preserve fine-grained information during the transfer of global structure from the source domain to the target domain, task-specific knowledge can be leveraged to explore the intrinsic and consistent class structure of the target domain data, which enhance the reliability and generalization during the transfer process. To this end, we employ a hybrid neural structure to extract multiple representations from images, together with subdomain adaptation, to capture the fine-grained information for each disease class. As the images of target domain are not labeled, we generate pseudo labels for the target data by adopting a self-training strategy. However, the generated pseudo label for the target domain may be incorrect, and using this incorrect label may cause a negative transfer of irrelevant knowledge. Hence, we utilize probabilistic prediction (i.e., soft prediction) to alleviate the negative effects of pseudo labeling. An auxiliary regularization based on semisupervised learning is further introduced to drive decision boundaries of different disease categories to the areas with low density, which accordingly boosts the discriminative ability of our transfer model.

Concretely, our proposed framework, referred to as Multi-Representation Subdomain Adaptation Network with Uncertainty Regularization for Cross-Species Plant Disease Classification (MSUN), first reduces the discrepancies between 2 domains while addressing uncertainties within and across domains. A hybrid neural structure is exploited to capture more detailed information by learning multiple domain-invariant representations. To retain more semantic information, we apply local maximum mean discrepancy (LMMD) to align the feature distribution of the relevant subdomains. Auxiliary uncertainty regularization is then introduced to counteract the unreliability problem in the adaptive process due to the accumulation of misleading information in incorrect labels and the reduced confidence in predicting low-quality plant images. Furthermore, we choose a nonadversarial approach to implement our model, which is very simple but effective.

Our contributions are summarized as follows:

1. We propose a nonadversarial UDA approach to address the uncertainty in classifying various diseases among species to alleviate the discrepancy of feature distribution between source and target domains.

2. We propose to capture multiple domain-invariant representations of diseased plant leaves, and preserve semantic information by designing an Inception Adaptation framework. This largely aids in improving the classification accuracy of cross-species plant diseases, especially on plant images collected from a field environment.

3. We introduce an additional regularization to avoid the potential adverse adaptation caused by the cluttered background in the collected plant images, thus enhancing the reliability of the domain transfer with excellent generalization which is verified by laboratory-controlled and field datasets.

## Related Work

### Unsupervised domain adaptation

In order to reduce the burden of labeling target data in a computer vision task, many UDA methods [19–21] have been proposed. Recently, adaptive methods in the field of deep networks have been extensively studied [22,23]. Compared with the shallow adaptive methods, deep network achieves better performance. Several other studies have also been presented to improve the model performance, which learn the domain-invariant features by designing a discriminator [24–27]. There are a few studies on applying UDA methods to the field of plant and agriculture. Ayalew et al. [28] propose the use of a UDA method to transfer the density map estimation for counting plant organs. Giuffrida et al. [29] present the model used for estimating leaves from a specific dataset to an unknown dataset based on an unsupervised adversarial adaptation mechanism. Both of these 2 methods directly use the UDA method for the task of plant or agriculture. Although the UDA methods have been shown to improve classification performance, these methods assume that the prediction error is limited by the divergence of the feature distribution. No consideration is given to the relationship between target samples and classification decision boundaries, which may degrade the model’s performance due to adverse transfer result from irrelevant knowledge. Therefore, we consider the underlying class-level data structure of the target domain rather than just aligning the global feature distribution. Our work focuses on capturing the fine-grained characteristic existing in plant disease classification and retaining the semantic information by aligning class conditional distributions in the adaptive process.

### Semantic alignment

Numerous nonadversarial and adversarial methods have achieved global alignment in UDA. However, global domain alignment reduces the difference between the source domain and target domain by learning domain-invariant features while ignoring their class-level multimodal structures. This makes the domain adaptation at risk of semantic misalignment. To mitigate this issue, more attention is paid to semantic alignment, which is beneficial to combine fine-grained class-level structures in various tasks appropriately. Wang et al. [30] propose a self-adaptive reweighted adversarial domain adaptation approach, which generates features with better interclass separation and intraclass compactness to realize class-level alignment. By learning fine-grained information adaptively in subdomains, deep subdomain adaptation network (DSAN) [31] solves the problem of image classification. In transferable attention for domain adaptation [32], the correctness of image classification is improved by considering the transferability of different images and focusing the adaptive model on the transferable regions or images. Yan et al. [13] applied a mixed subdomain adaptive method to classify plant disease severity across species, promoting the computer-aided diagnosis of plant diseases. In conditional adversarial domain adaptation [33], the discriminative information conveyed in the classifier prediction is used to assist the antagonism adaptation to realize semantic alignment. Despite the efficacy of existing semantic alignment methods, these methods failed to take into account the important difference between 2 domains, where transfer learning from one to another is usually unsatisfactory. In our work, we aim to learn from the source domain the knowledge of multiple representations and adopt this knowledge when the subdomains are aligned. In this way, we effectively address the problem of negative transfer caused by the large discrepancy between plant data from the laboratory and the field environment.

## Materials and Method

### MSUN method

**Fig. 2.**
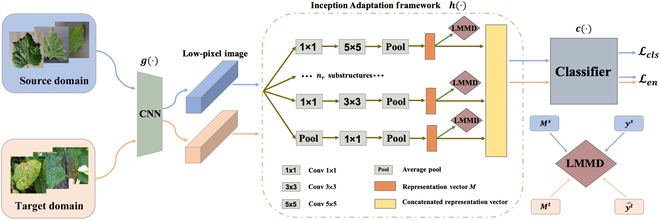
(Left) Workflow of the proposed MSUN. The blue and light orange flow denote operations in the source and target domains, respectively. (Right) Alignment using LMMD.

The proposed method MSUN is composed of a feature generator g(·), a classifier c(·), and an Inception Adaptation Network module h(·), which learns multirepresentation meaningful disease-related features (as shown in Fig. 2). The Inception Adaptation Network module includes multiple substructures combining neural networks with kernels of varied sizes, which captures the useful multiscale features more effectively. To preserve more semantics, the LMMD metric is adopted to align the feature distributions of substructures. An auxiliary uncertainty regularization $\;L_{\mathrm{en}}$ is exploited to pull decision boundaries away from areas with high density, which thus enforces the adaptation to be more consistent and discriminative.

In our work, we employ UDA to bridge the gap between laboratory and field environments, where 2 datasets collected from different conditions are given. One is the source domain $D_{s}={\left\{(x_{i}^{s},y_{i}^{s})\right\}}_{i=1}^{n_{s}}$ containing *n_s_* corresponding samples $x_{i}^{s}$ labeled by $y_{i}^{s}\in {\mathbb{R}}^{C}$, and the other is the target domain $D_{t}={\left\{x_{j}^{t}\right\}}_{j=1}^{n_{t}}$ containing *n_t_* unlabeled samples $x_{j}^{t}$. $D_{s}\;$ and $D_{t}$ are obtained by sampling from different data distributions *p* and *q* where *p* ≠ *q*. Suppose that the source domain has the same tasks as the target domain. The goal is to develop a deep neural network f: X → Y, which predicts the labels of samples from the target domain more accurately. In LMMD, *M^s^* and *M^t^* represent the multirepresentation vectors in the source and target domains, respectively. *y^S^* and $\hat{y^{t}}$ are the ground-truth label and the predicted label, respectively.

MSUN aligns the subdomain distribution of multiple representations. The proposed network involves a CNN *g*(·), multirepresentation extraction module *h*(·), and a classifier *c*(·).

#### Multirepresentation module

Much has been achieved in recent years with deep transfer networks. Still, these methods that we call single-representation adaptation methods only align the distribution of representations extracted by a single structure. However, the single-representation adaptation methods focus on the partial information of the image samples, where such information retained during the transfer might be insufficient to represent the features, and the model cannot thus be generalized well for diverse scenarios. In our case, images collected from the laboratory and field differ largely. For example, the laboratory image contains only a diseased leaf, while the field image contains other information such as the fruit next to the leaf or some scattered twigs on the ground. Based on the observation of our experiments, the single representation method achieves unsatisfying classification performance when adapted to the target field scenario. To this end, we make the network learn the overall structure of the features by learning multirepresentation information from the source domain. In this way, it will ignore information that would affect its judgement when the network is applied to the target domain

Since it is time-consuming to train several different CNNs to learn several different domain-invariant representations, inspired by the work in [34], we adopt a hybrid structure to learn multirepresentation information. The hybrid structure is divided into 3 parts: *g*(·), *h*(·), and *c*(·). We use the first part of the CNN *g*(·) to convert high-pixel images to low-pixel images, where a high-pixel image is the original input image, and a low-pixel image is an image containing easily expressed and generalized features. The second part is *h*(·), designed to extract multiple representations from low-pixel images. The third part is the classifier *c*(·), which predicts the labels. The second part *h*(·) consists of several mutually distinct substructures *h*_1_(·), …, *h_n_r__*(·), where *n_r_* is the number of substructures. It is known that the larger the convolution kernel, the larger its receptive field, and thus the better the extracted features. However, large convolutional kernels can lead to a considerable increase in computation and hence a decrease in performance. Therefore, we consider 3 types of convolutional kernels, 1 × 1, 3 × 3, and 5 × 5, for the substructure.

Each substructure consists of different convolution kernels with pooling layers depending on extracted features. Since the background of plant disease images collected in the field environment contains irrelevant information, it is necessary to focus on both the detail of the disease on the leaf and the whole leaf in its environment. Based on the above rationale, our multifeature extraction module uses a total of 4 substructures. Substructure1 is conv1 × 1, conv5 × 5, and average pool. After the operation of substructure1 is completed, the approximate positions where the leaf blade in an image are highlighted. To better determine the shape, texture, and color of the leaf, we design substructure2 as conv1 × 1, conv 3 × 3, conv 3 × 3, and average pool. The first 3 × 3 convolution kernel achieves an image-sharpening effect, and the second 3 × 3 convolution kernel produces brighter edges. After the results of 2 kernels have been superimposed, and the details of the leaf are extracted. Substructure3 is conv1 × 1 and average pool, which is responsible for the feature communication of the channels of the low-pixel images acquired by the network *g*(·). The relationship between the leaves and the background environment in the low-pixel image is preserved as much as possible. This avoids too much loss in accuracy due to the operation of the remaining substructures. Due to the need to understand the global information of the leaf in the entire captured image, substructure4 contains average pool, conv1 × 1, and average pool. Average pooling is performed first to preserve the features of the overall data in the image and better highlight the background information. This is followed by a 1 × 1 convolution to compress the feature map and extract features twice.

The above 4 substructures effectively solves the problem of large differences between the source and target domains due to the environment in which the images are captured. We obtain multiple representations (*h*_1_ ∘ *g*)(**X**), …, (*h_n_r__* ∘ *g*)(**X**) with a hybrid module *h*(·). Minimizing differences in distributions based on multiple representations for our adaptation tasks is achieved by

$$\min_{f}\sum_{i}^{n_{r}}\hat{d}(\left(h_{i}\circ g\right)\left(X_{s}\right),\left(h_{i}\circ g\right)\left(X_{t}\right))$$ (1)

where **X** denotes the set of *x* and $\hat{d}(\cdot ,\cdot )$ stands for an estimate of the difference between the 2 distributions. The classification task is achieved by placing the concatenated vector into a classifier *c*(·) which contains a fully connected layer and a softmax layer. Recombining multiple representations is achieved by the fully connected layer, and the predicted labels are generated by the softmax layer. Finally, the neural network *y* = *f*(**x**) embedded in the hybrid structure is formulated as

$$y=f(x)=c([(h_{1}\circ g)(X);\ldots ;(h_{n_{r}}\circ g)(X)]).$$ (2)

#### Subdomain adaptation module

It is necessary to select the appropriate distance metric to measure Eq. 1. Maximum mean discrepancy (MMD) is widely used to measure discrepancies in marginal distributions, i.e.,

$${\hat{d}}_{H}(X_{s},X_{t})={\left\|\frac{1}{n_{s}}\sum_{x_{i}\in D_{X^{s}}}\phi \left(x_{i}\right)-\frac{1}{n_{t}}\sum_{x_{j}\in D_{X^{t}}}\phi \left(x_{j}\right)\right\|}_{H}^{2}$$ (3)

where H is the reproducing Hilbert kernel assigned to the characteristic kernel k. *ϕ(·)* denotes a mapping function and kernel k is implemented using *k*(**x***^s^*, **x***^t^*) = ⟨ *ϕ*(**x***^s^*), *ϕ*(**x***^t^*)⟩. In practice, the empirical kernel mean embedding is used to estimate the squared value of the MMD, i.e.,

$$\begin{array}{c} \begin{array}{l} {\hat{d}}_{H}(p,q)={\left|\left|\frac{1}{n_{s}}\sum_{x_{i}\in D_{s}}\phi \left(x_{i}\right)-\frac{1}{n_{t}}\sum_{x_{j}\in D_{t}}\phi \left(x_{j}\right)\right|\right|}_{H}^{2}\\ =\frac{1}{n_{s}^{2}}\sum_{i=1}^{n_{s}}\sum_{j=1}^{n_{s}}k(x_{i}^{s},x_{j}^{s})+\frac{1}{n_{t}^{2}}\sum_{i=1}^{n_{t}}\sum_{j=1}^{n_{t}}k(x_{i}^{t},x_{j}^{t})\\ -\frac{2}{n_{s}n_{t}}\sum_{i=1}^{n_{s}}\sum_{j=1}^{n_{t}}k(x_{i}^{s},x_{j}^{t}). \end{array} \end{array}$$ (4)

**Fig. 3.**
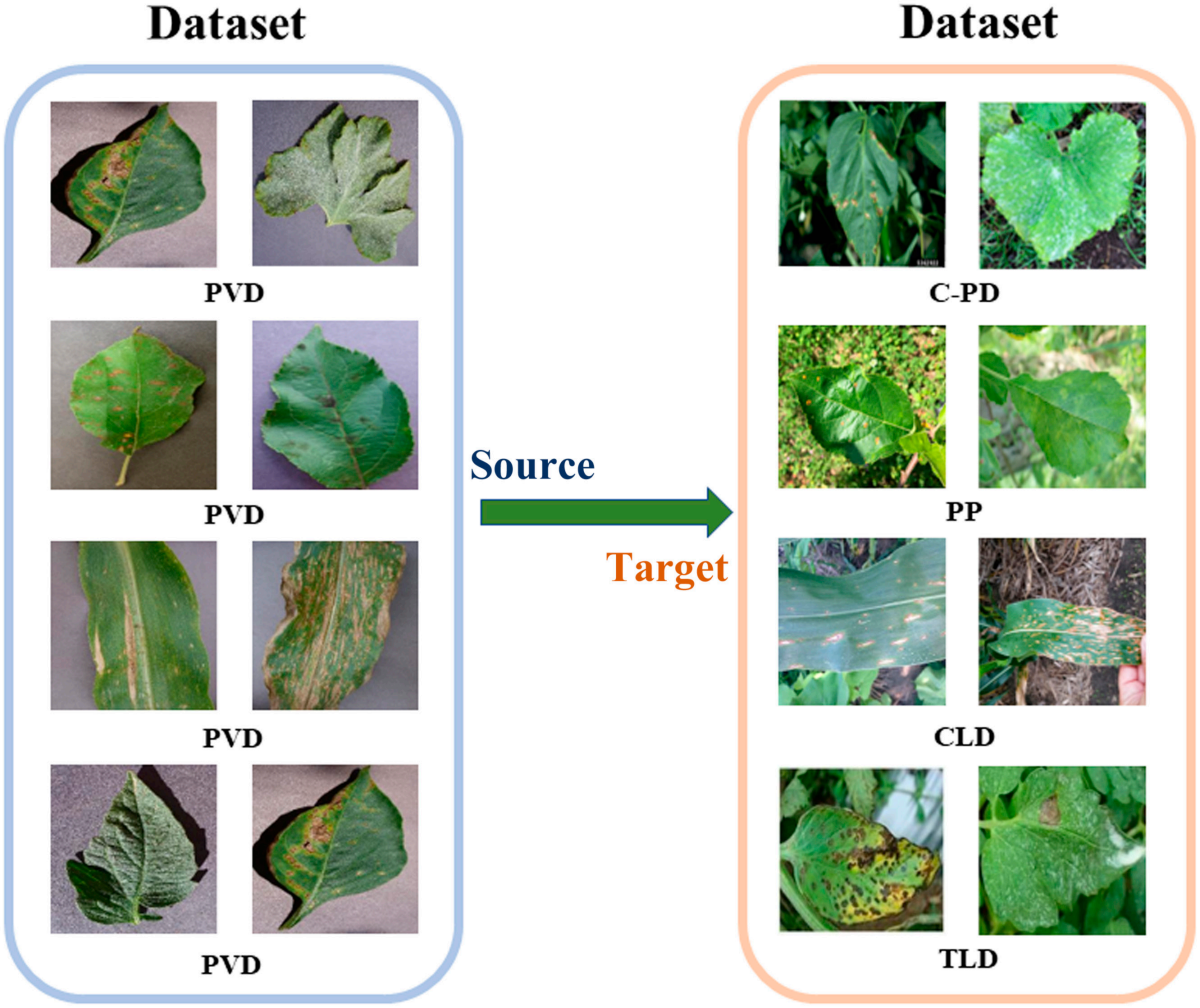
Domain adaptation processes on 4 experiments: (Left) Source domain dataset. (Right) Target domain dataset.

Various plant diseases across species tend to have high intraclass variation and high interclass similarity. While MMD focuses only on aligning the global source and target distributions, it does not consider the relationships between subdomains within different domains of the same category. After global domain adaptation, source and target domains contain nearly the identical distribution. However, the features belonging to the different subdomains (disease species) are too similar to be easily classified. The poor alignment of the global distribution can be visualized in Fig. 3. As a result, MMD fails to perform well in our work. Furthermore, the same plant species suffering from the same disease may have different growing environments or be infected by the disease at different times. This leads to a more obvious variation in the disease status presented by the plant leaves, which explains the high intraclass variation. Yet, symptoms of the same disease are similar in different plant species, which indicates high interclass similarity. This results in a tendency for intraclass distances to be greater than interclass distances, resulting in a negative transfer of irrelevant knowledge.

It is expected that samples from the same category should be clustered together. However, in the embedding feature space, clusters belonging to different categories tend to overlap, introducing difficulties to the classification. In addition, predicting uncertainty incurred by overlapping ambiguous clustering is exacerbated when only the alignment of the global source and target distributions are considered. Subdomain alignment takes into account discrepancies in the distribution of the subdomains, allowing the network to capture fine-grained information more effectively, thus improving the classification performance of the classifier on subdomains. According to [35], samples from the same category should be located in the same subspace, even if they belong to different domains. Thus, we considered exploiting the above relationships between samples to divide the source and target domains into multiple subdomains containing samples of the same class. Unfortunately, the sample labels in the target domain are unavailable. To address this, we first trained the model using data with labels from the source domain. The trained model is then utilized to predict the unlabeled data from the target domain, where the predictions are considered as pseudo-labels. To achieve subdomain alignment, we split $D_{s}$ and $D_{t}$ into *C* subdomains $D_{s}^{\left(c\right)}$ and $D_{t}^{\left(c\right)}$ based on the category, where *c* ∈ {1, 2, …, *C*} is the class label. *p*^(*c*)^ and *q*^(*c*)^ respectively denote the distributions of $D_{s}^{\left(c\right)}$ and $D_{t}^{\left(c\right)}$.

The purpose of subdomain adaptation is to align the distribution of subdomains with identically labeled samples. Motivated by the work in [27], we adopt LMMD for estimating the difference in distribution between subdomains, i.e.,

$$d_{H}(p,q)\overset{\Delta }{=}E_{c}{\left\|E_{p^{\left(c\right)}}\left[\phi \left(x^{s}\right)\right]-E_{q^{\left(c\right)}}\left[\phi \left(x^{t}\right)\right]\right\|}_{H}^{2},$$ (5)

where **x***^s^* and **x***^t^* respectively denote instances from $D_{s}$ and $D_{t}$, and *p*^(*c*)^ and *q*^(*c*)^ respectively represent the distributions of $D_{s}^{\left(c\right)}$ and $D_{t}^{\left(c\right)}$. In our work, LMMD is minimized in training so that the distribution distance of related subdomains within the same category is reduced, enabling the network to learn more fine-grained information. Assume that each sample belongs to each category according to the weights *w^c^*. The unbiased estimator of Eq. 3 is then denoted as

$${\hat{d}}_{H}(p,q)=\frac{1}{C}\sum_{c=1}^{C}{\left\|\sum_{x_{i}^{s}\in D_{s}}w_{i}^{\mathrm{sc}}\phi \left(x_{i}^{s}\right)-\sum_{x_{j}^{t}\in D_{t}}w_{j}^{\mathrm{tc}}\phi \left(x_{j}^{t}\right)\right\|}_{H}^{2},$$ (6)

where $w_{i}^{\mathrm{sc}}$ and $w_{j}^{\mathrm{tc}}$ are respectively the weight of $X_{i}^{S}$ and $x_{j}^{t}$ belonging to the same class *c*. Both $\sum_{i=1}^{n_{s}}\;w_{i}^{\mathrm{sc}}$ and $\sum_{j=1}^{n_{t}}\;w_{j}^{\mathrm{tc}}$ have the value 1, and $\sum_{x_{i}\in D}\;w_{i}^{c}\phi \left(x_{i}\right)$ denotes the weighted sum of category *c*. The $w_{i}^{c}$ of sample **X***_i_* is given by

$$w_{i}^{c}=\frac{y_{\mathrm{ic}}}{\sum_{(x_{j},y_{j})\in D}y_{\mathrm{jc}}},$$ (7)

where *y_ic_* indicates the *c*th term of the vector **y***_i_*. For the source domain samples, we use the true labels. In contrast, for the unlabeled target domain samples, we use the output of the neural network ${\hat{y}}_{i}=f\left(x_{i}\right)$ since it is a probability distribution that describes well the probability of assigning **X***_i_* to each *C* class.

We apply LMMD to the representation vector extracted from each substructure in the multirepresentation module *h*(·). Thus, Eq. 6 is reformulated as

$${\hat{d}}_{H}\left(p,q\right)=\frac{1}{C}\sum_{c=1}^{C}{\left|\left|\frac{1}{n_{s}}\sum_{x_{i}^{s}\in D_{s}}w_{i}^{\mathrm{sc}}\phi \left(x_{i}^{s}\right)-\frac{1}{n_{t}}\sum_{x_{j}^{t}\in D_{t}}w_{j}^{\mathrm{tc}}\phi \left(x_{j}^{t}\right)\right|\right|}_{H}^{2}.$$ (8)

Since we cannot calculate *ϕ*(·) directly, we replace Eq. 8 with

$$\begin{array}{c} \begin{array}{l} {\hat{d}}_{l}\left(p,q\right)=\frac{1}{C}\sum_{c=1}^{C}\left[\frac{1}{n_{s}^{{\left(c\right)}^{2}}}\sum_{i=1}^{n_{s}}\sum_{j=1}^{n_{s}}w_{i}^{\mathrm{sc}}w_{j}^{\mathrm{sc}}k(M_{i}^{s},M_{j}^{s})\right.\\ +\frac{1}{n_{t}^{{\left(c\right)}^{2}}}\sum_{i=1}^{n_{t}}\sum_{j=1}^{n_{t}}w_{i}^{\mathrm{tc}}w_{j}^{\mathrm{tc}}k(M_{i}^{t},M_{j}^{t})\\ \left.-\frac{2}{n_{s}^{\left(c\right)}n_{t}^{\left(c\right)}}\sum_{i=1}^{n_{s}}\sum_{j=1}^{n_{t}}w_{i}^{\mathrm{sc}}w_{j}^{\mathrm{tc}}k(M_{i}^{s},M_{j}^{t})\right] \end{array} \end{array}$$ (9)

where *M* is the representation vector and *p* and *q* respectively denote the distribution of the source and target domains. This is followed by applying Eq. 9 to measure Eq. 1. By minimizing Eq. 9, the source and target domains are well aligned.

#### Auxiliary uncertainty regularization

Uncertainties from both domains are greatly mitigated by using multirepresentation subdomain adaptation. However, by observing the class clusters during adaptation, a number of feature points are still present near the decision boundary. This is because the fine-grained characteristic of plant disease and large domain discrepancy between the scenarios of laboratory and field influence the pseudo labeling of target domain seriously, which may bring extra uncertainty to the adaptation process. Inspired by the semisupervised learning, we employ the entropy minimization technique to deal with such uncertainty as follows.

In semisupervised learning, abundant unlabeled target data is capable of encouraging the decision boundaries of classifiers to go through the areas which includes low-density data points [36]. This is referred to as the clustering assumption [37], which has also been applied in domain adaptation [38]. In this case, we employ entropy minimization to pull decision boundaries away from the regions with high density, where the uncertainty of embedded disease-related features is alleviated. By driving the boundaries to the area with low density of the target domain, the discriminative ability is further strengthened during the transfer. This behavior is achieved via entropy minimization with respect to the target distribution using

$$L_{\mathrm{en}}\left(f\right)=-E_{x\%D_{t}}\left[f{\left(x\right)}^{⊤}\ln\left(f\left(x\right)\right)\right],$$ (10)

where *f*(*x*) denotes the predicted class score of the classifier. In our work, to surpass such uncertainty and acquire more discriminative ability, the unlabeled target data are fully exploited. Intuitively, minimizing the entropy enables the classifier to be confident in the unlabeled target domain data, which drives the decision boundaries away from the data and thus tackled the uncertainty due to the pseudo-labeling.

#### Multirepresentation subdomain adaptation network

In this work, we propose to learn multiple representations and embed an effective nonadversarial loss LMMD, while introducing auxiliary uncertainty regularization. The loss of our network is

$$\begin{array}{c} \min_{f}\frac{1}{n_{s}}\sum_{i=1}^{n_{s}}J(f(x_{i}^{s}),y_{i}^{s})+\lambda \sum_{i}^{n_{r}}\hat{d}((h_{i}\circ g)(X_{s}),\\ \;(h_{i}\circ g)(X_{t}))+\gamma L_{\mathrm{en}}, \end{array}$$ (11)

where *J*(·, ·) denotes the cross-entropy loss function (classification loss), $\hat{d}(\cdot ,\cdot )$ represents domain adaptation loss given by Eq. 6, and $L_{\mathrm{en}}$ is entropy minimization constraint. *λ* and *γ* are the trade-off parameters for each of the 2 proposed components. Note that the approach in aligning source and target feature distributions for domain adaptation is based on a theoretical analysis of domain adaptation [39–41]. The detailed training procedure for MSUN is given in Algorithm 1.

Algorithm 1. Training of MSUN

**Table:** 

**Input:** Source domain $S={\left\{(x_{i}^{s},y_{i}^{s})\right\}}_{i=1}^{n_{s}}$, target domain $\;T={\left\{\left(x_{i}^{t}\right)\right\}}_{i=1}^{n_{t}}$ **Output:** Classifier *f_C_*(∙) **Begin:** 1: Load the pre-trained model 2: **for** epoch **do** 3: **for** minibatch **do** 4: # Obtain target domain pseudo-labels 5: ${\hat{Y}}^{T}\leftarrow {\left\{(x_{i},f_{C}(x_{i}^{t}))\right\}}_{i=1}^{n_{t}}$, where $x_{i}^{t}\in T$ 6: # Convert high-pixel images to low-pixel ones 7: *g^s^*, *g^t^* ← *S*, *T* , using *g*(·) 8: # Obtain multi-representation 9: *M^s^*, *M^t^*← *g^s^*, *g^t^* , using *h*(·) 10: # Determine samples weights 11: Calculate $w_{i}^{\mathrm{sc}}$ and $w_{j}^{\mathrm{tc}}$ using Eq. 7 12: # Determine the loss of MSUN 13: Calculate $L_{C}(Y^{S},{\hat{Y}}^{S})$ 14: Calculate $L_{\mathrm{LMMD}}(M^{s},M^{t},Y^{S},{\hat{Y}}^{T})$ using Eq. 9 15: Calculate $L_{\mathrm{en}}(M^{s},M^{t})$ using Eq. 10 16: Calculate total loss $L_{\mathrm{MSUN}}$ using Eq. 11 17: Update parameters by back propagation $L_{\mathrm{MSUN}}$ 18: **end for** 19: **end for** **End**

### Datasets

To evaluate the performance of the proposed MSUN, we conducted 4 sets of experiments involving 5 datasets: PlantVillage [42], PlantDoc [43], Plant-Pathology [44], Corn-Leaf-Diseases, and Tomato-Leaf-Diseases. The PlantVillage dataset (PVD) is consistently used for the source domain in all experiments as it is collected in the laboratory environment and thus contains a plain background.

PVD, which comprises images captured in a laboratory environment, is a large publicly available dataset for plant disease detection. It contains a total of 54,306 images of plant leaves, with a total of 38 class labels. We use PVD as the source domain dataset due to its abundance of samples and its widespread use in plant disease detection work. Depending on the transfer task and the target dataset, we select the appropriate category in PVD. There are 2 versions of the PlantDoc dataset, cropped and uncropped. The images in the uncropped version may contain more than one region of interest (i.e., diseased or healthy leaves). In the cropped version (C-PD), an annotation box is added around the region of interest of an uncropped image and then cropped. The cropped version which includes 8,897 images of plant leaves of 27 classes is chosen for our classification task. Since the annotation boxes in the PlantDoc dataset are not always correct, we removed images with obvious errors (i.e., annotation boxes labeled on apples instead of apple disease leaves) and ended up with a selection of 5,601 images. The PVD-PD experiments are based on the PVD and C-PD datasets. Since the PVD-PD experiment involves many categories and fewer images in the target domain, to avoid overfitting, we selected 20% of the samples from the original PVD dataset as the source domain dataset for this experiment. Table 1 shows the categories and specific amounts of the dataset.

**Table 1.** Image categories and the number of images used in the PVD-PD experiment.

Class		PVD	C-PD
Apple	Scab	251	134
	Cedar rust	219	138
	Healthy	397	241
Blueberry	Healthy	762	546
Cherry	Healthy	386	275
Corn	Gray leaf spot	153	67
	Leaf blight	286	119
	Rust	326	209
Grape	Healthy	383	112
	Leaf black rot	231	81
Peach	Healthy	156	65
Pepper bell	Healthy	239	151
	Spot	395	234
Potato	Early blight	269	172
	Late blight	339	203
Raspberry	Healthy	159	61
Soybean	Healthy	428	216
Squash	Powdery mildew leaf	440	214
Strawberry	Healthy	259	103
Tomato	Early blight leaf	480	217
	Septoria leaf spot	540	186
	Healthy	508	343
	Leaf bacterial spot	458	219
	Leaf late blight	428	215
	Leaf late blight	525	390
	Leaf yellow virus	331	211
	Mold leaf	685	479
Total		10,033	5,601

The Plant-Pathology dataset (PPD) consists of a total of 4,900 images of apple leaves with 4 main classes (i.e., healthy, scab, rust, and multiple diseases). For the PVD-PP experiments, 3 categories are selected, and the specific categories and numbers of the dataset are given in Table 2.

**Table 2.** Image categories and the number of images used in the PVD-PP experiment.

Class	PVD	PPD
Apple leaf scab	504	1,604
Apple leaf cedar rust	223	370
Apple leaf healthy	380	1,411
Total	1,107	3,385

To validate the effectiveness of our proposed method for different diseases and datasets, we obtained additional datasets of corn and tomato leaf diseases from the Kaggle website. The Corn-Leaf-Diseases dataset (CLD) containing 4,188 images comprises 4 classes. In the PVD-CLD experiment, we use the CLD dataset as the target domain. Table 3 shows the categories and number of images in the datasets.

**Table 3.** Image categories and the number of images used in the PVD-CLD experiment.

Class	PVD	CLD
Corn gray leaf spot	411	1,604
Corn leaf blight	788	370
Corn leaf healthy	930	1,162
Corn leaf rust	954	1,411
Total	3,083	4,547

The Tomato-Leaf-Diseases dataset (TLD) containing 2,974 images consists of 5 classes. Table 4 shows the categories of the datasets involved in the PVD-TLD experiments as well as their number of images.

**Table 4.** Image categories and the number of images used in the PVD-TLD experiment.

Class	PVD	TLD
Tomato leaf bacterial spot	1,020	704
Tomato leaf healthy	1,272	678
Tomato leaf late blight	1,528	561
Tomato mold leaf	762	428
Tomato Septoria leaf spot	1,417	537
Total	5,999	2,908

The domain adaptation process for the 4 experiments based on the 5 datasets is illustrated in Fig. 3. Note that all 4 datasets were captured in the field, except for the images in the PVD, which were captured in a laboratory environment. All images in the PVD dataset are used for the remaining 3 experiments, except for the PVD-PD experiment, where we select only 20% of the images in the source domain dataset PVD.

## Experiments and Results

We compare our model MSUN with several prevalent approaches, including deep adaptation network (DAN) [45], Deep CORAL (D-CORAL) [46], domain adversarial neural network (DANN) [28], dynamic adversarial adaptation network (DAAN) [27], DSAN [31], and multirepresentation adaptation network (MRAN) [34]. To highlight the effectiveness of using domain adaptation techniques, we also include the model without domain adaption, i.e., the Baseline (w/o DA), for comparative experiments. The Baseline and all domain adaptation methods use the ResNet [47] as the backbone for achieving fair comparative results.

### Implementation details

The detailed training procedure for MSUN is given in Algorithm 1. The computers used in our experiments are configured as follows: NVIDIA GeForce GTX 3080TI, 24GB RAM, i7-11700k. The deep learning framework used in this work is Pytorch 1.9. Due to the unsupervised task, we regard the labeled data as the source domain and the unlabeled data as the target domain. For each dataset the images are first resized to 256*256 and then randomly cropped to 224*224. We also augment the data by random flipping. We adopt ResNet (50 layers) to learn the transferable depth representation, with the activation of the last feature layer pool5 as the image representation of the baseline. Since a large amount of labeled data is required when training deep networks, which is difficult for many domain adaptive applications, we start with CNN pretraining of ImageNet data. We then fine-tune the pretrained model and train the classifier layer by back-propagation.

We set the learning rate of the classifier to 10 times that of the other layers, as it is trained from scratch. The training of MSUN follows the standard minibatch stochastic gradient descent algorithm with a momentum of 0.9. To eliminate bias caused by the domain size in each small batch, sampling is based on the same amount of source and target domain data. We train a total of 200 epochs, and the whole network is optimized together with the losses presented in Eq. 11. To suppress noisy activations during the early stages of training, we gradually alter the adaptive factor *λ* from 0 to 1 using a progressive schedule, $\lambda_{p}=\frac{2}{\exp\left(-\alpha p\right)}-1$, and *α* is set to 10 in our work [29]. *γ* is optimized through a grid search of predefined sets {0, 0.01, 0.02, 0.03, 0.05, 0.1, 0.2, 0.5}.

### Results analysis

In plant disease transfer detection tasks, MSUN performs largely better than all related methods as shown in Table 5. The C-PD dataset contains a total of 27 classes, consisting of 13 plant species. The C-PD dataset covers a wide range of disease categories and plant species and was created in a field environment, which is quite different from the images captured in the laboratory. Under this setting, the large interdomain variation could make the transfer not particularly satisfactory. However, our proposed MSUN model provides a strong focus on the problem of weak interdomain associations, so that MSUN still yields promising results. Compared to other methods, our model substantially improves the average accuracy by 10% to 56.06%.

**Table 5.** Accuracy (%) results of the PVD-PD experiment.

Method	Apple	Corn	Grape	Pepper_bell	Potato	Tomato	Average
Baseline (w/o DA)	67.38	50.07	71.36	84.19	50.34	30.17	30.78
DAAN	68.64	48.81	63.93	87.79	63.66	39.82	33.08
D-Coral	69.06	49.11	79.79	86.75	66.06	43.32	33.37
DAN	70.29	52.15	78.24	90.91	64.72	46.23	33.79
DANN	69.63	48.11	83.41	90.90	62.33	49.51	39.71
MRAN	68.98	49.87	82.38	92.46	57.82	49.34	39.84
DSAN	65.51	51.39	79.79	90.91	64.19	45.75	42.87
MSUN	71.45	56.46	85.49	93.49	67.42	48.67	56.06

Tables 6–8 provide specific results for the PVD-PP, PVD-CLD, and PVD-TLD experiments, respectively. From the experiments with single plant species (i.e., PPD, CLD, TLD, and each plant species in C-PD), we observe that the transfer of single species is generally better compared to the transfer of multiple plant species together in C-PD. Note that it is easy to confuse different plant species suffering from the same disease during transfer due to the similarity in the manifestation of the same disease on the leaves of different plant species. However, in the case of single plant species transfer, the issue of blurred boundaries between classes in the domain is alleviated. Furthermore, the multirepresentation and subdomain adaptation modules in MSUN effectively address the problem of interclass similarity and large intraclass variation, enabling our model to achieve better results in the transfer of a single plant species as well. Overall, MSUN achieves excellent results in both multiple- and single-plant- species tasks, demonstrating its effectiveness in mitigating various domain shifts.

**Table 6.** Accuracy (%) results of the PVD-PP experiment.

Method	Gray spot	Rust	Healthy	Average
Baseline (w/o DA)	79.54	24.10	11.02	39.27
DAN	89.02	34.99	11.84	48.89
DANN	85.12	30.26	28.71	50.04
D-Coral	77.56	33.42	48.05	52.41
DAAN	66.16	55.81	36.64	53.75
DSAN	83.96	35.42	51.24	56.41
MRAN	81.28	52.57	70.87	67.45
MSUN	87.32	57.71	73.86	72.31

**Table 7.** Accuracy (%) results of the PVD-CLD experiment.

Method	Gray spot	Blight	Healthy	Rust	Average
Baseline (w/o DA)	61.49	86.75	79.05	99.18	75.96
DAN	68.29	91.50	81.33	100.0	87.89
DANN	76.66	91.73	82.64	100.0	89.47
D-Coral	79.44	91.88	82.64	100.0	89.90
DAAN	78.57	92.27	84.29	100.0	90.35
DSAN	81.71	91.58	83.42	100.0	90.69
MRAN	79.22	92.05	83.61	100.0	91.06
MSUN	80.09	93.81	84.77	100.0	96.78

**Table 8.** Accuracy (%) results of the PVD-TLD experiment.

Method	Bacterial spot	Healthy	Late blight	Mold leaf	Septoria spot	Accuracy
ResNet (w/o DA)	5.34	70.61	1.25	25.03	6.36	21.46
DSAN	10.51	92.03	1.64	29.61	0.29	27.57
DANN	8.24	86.92	3.50	38.73	4.13	28.71
DAAN	10.23	83.75	3.97	33.15	5.16	30.96
D-Coral	12.50	85.81	1.64	37.43	18.73	32.31
DAN	12.22	83.41	0.93	38.18	67.11	42.80
MRAN	18.47	75.92	2.11	33.15	78.64	44.86
MSUN	29.27	78.67	3.28	32.77	80.79	50.58

As can be observed from the experimental results, our proposed MSUN method not only performs well in the disease classification transfer of a single plant species (as shown in Tables 6–8) but also achieves surprisingly good results in the disease classification transfer of plants across species. This is attributed to the design of our model. The multirepresentation module learns more information within an image, which is good at alleviating the problem of large interdomain disparity and large intraclass discrepancy within a domain. Subdomain adaptation focuses more on fine-grained information and enables semantic alignment. This further reduces the problem of large intraclass discrepancy within a domain and fuzzy boundary between classes in a domain. The auxiliary uncertainty regularization introduced by the model is a good solution to the problem of uncertainty in the transfer process caused by pseudo-labeling. These efforts enable MSUN to achieve excellent results on 4 publicly available datasets.

## Discussion

### Ablation study

To better evaluate the validity of the MSUN modules, we performed ablation study on all 4 in-the-field datasets. There are 3 main modules in the proposed MSUN, i.e., multiple representation module (*MUL-REP*), subdomain adaptation module with LMMD (*LMMD*), and uncertainty regularization with entropy minimization constraint (*EN*). We compared the accuracy by adding the 3 modules into the baseline gradually, and the results of different modules are shown in Table 9.

**Table 9.** Ablation study on 4 datasets (✓ denotes that the modules are included).

Dataset	Baseline	*MUL-REP*	*LMMD*	*EN*	Accuracy (%)
C-PD	✓				30.78
	✓	✓			41.26
	✓		✓		43.59
	✓	✓	✓		51.65
	✓	✓	✓	✓	56.06
PP	✓				39.27
	✓	✓			65.61
	✓		✓		58.47
	✓	✓	✓		69.15
	✓	✓	✓	✓	72.31
CLD	✓				75.96
	✓	✓			90.05
	✓		✓		86.68
	✓	✓	✓		92.37
	✓	✓	✓	✓	96.78
TLD	✓				21.46
	✓	✓			28.57
	✓		✓		37.12
	✓	✓	✓		46.45
	✓	✓	✓	✓	50.58

The problem of large interdomain distances is alleviated by learning more transferable information via the introduction of multirepresentation modules. The classification accuracy of the model with the multirepresentation module is greatly increased when compared to the baseline (without the multirepresentation module), especially for the datasets of PP and CLD (by 26.34% and 14.09%, respectively). The introduction of the subdomain adaptation module further increases the classification performance in all 4 datasets (by 10.39%, 3.54%, 2.32%, and 17.88%, respectively). This shows that subdomain adaptation is excellent in preserving fine-grained information, making semantic alignment rather than mere margin alignment possible. The proposed MSUN including all 3 modules achieves the accuracies of 56.06%, 72.31%, 96.78%, and 50.58%, which outperforms other models by large margins, demonstrating the effectiveness of the designed modules. We also observe that the accuracies of classification in the datasets of C-PD and TLD are relatively lower than PP and CLD. This could be due to the large variety of C-PD and TLD, where failure to consider the relationships between subdomains within different domains of the same disease category can lead to inaccurate classification.

### Parameter analysis

**Fig. 4.**
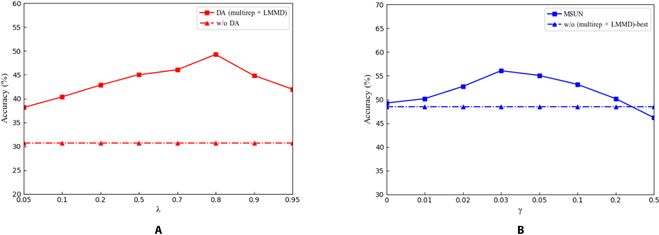
Sensitivity analysis of *λ* (A) and *γ* (B) for the MSUN optimization objective in Eq. 7 on the C-PD dataset.

We studied the sensitivity of *λ* and *γ* on the C-PD, and the results are demonstrated in Fig. 4. We first study the effect of λ representing the relative weight of multiple subdomain adaptation losses. The red dashed line in the figure denotes the performance of the baseline method (without DA). At this point, we set *γ* to 0. By varying *λ*∈{0.05, 0.1, 0.2, 0.5, 0.7, 0.8, 0.9, 0.95}, we notice that choosing a good trade-off (around 0.7) to weigh the importance of multiple representations and subdomain alignment leads to satisfactory adaptation results. This suggests that an appropriate trade-off effectively balances the impact of the 2 components added by MSUN on the model to enhance knowledge of transferability. We further studied the sensitivity of *γ*, which controls the magnitude of the effect of auxiliary uncertainty regularization on the target. In this case, we need to keep *λ* at the optimal value of 0.8. As *γ* takes on different values in the set {0, 0.01, 0.02, 0.03, 0.05, 0.1, 0.2, 0.5}, the accuracy of MSUN increases and then decreases, and appears as a bell-shaped curve. From the results, we are able to find a trade-off (around 0.1) that effectively improves adaptation performance.

### Feature visualization

**Fig. 5.**
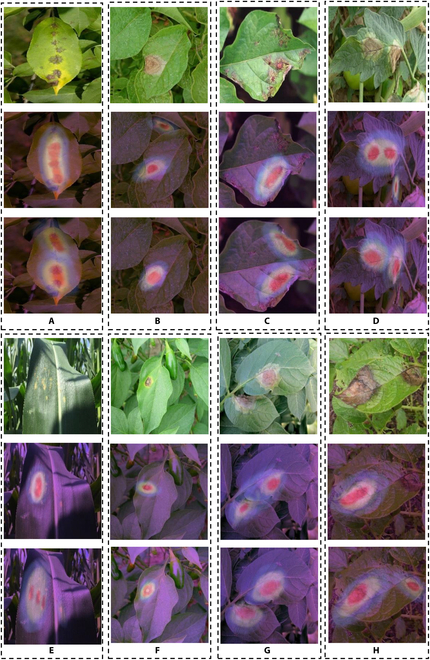
Visualization using Grad-CAM: (A) to (H) respectively correspond to apple scab leaf, potato leaf late blight, tomato early blight, tomato late blight diseases, corn leaf rust, pepper_bell leaf bacterial spot, potato leaf early blight, and potato leaf early blight. The upper image is the original image of the C-PD dataset, and the lower part is the maps of the network interest areas generated by Grad-CAM for DANN [43] and MSUN, respectively.

**Fig. 6.**
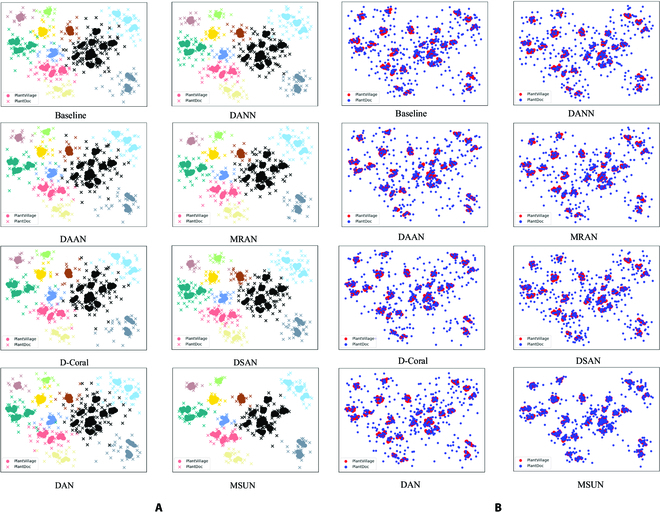
Visualizations of the learned representations using t-SNE on the C-PD dataset for Baseline [44], DAAN [33], D-Coral [42], DAN [17], DANN [43], MRAN [18], DSAN [27], and our MSUN, where subfigure (A) is colored based on the plant species, while subfigure (B) is colored based on the datasets.

To further demonstrate the validity of MSUN, we visualized the results of MSUN and other methods for comparison on the C-PD dataset using 2 commonly used visualization methods, i.e., gradient-weighted class activation mapping (Grad-CAM) [35] and t-distributed stochastic neighbor embedding (t-SNE) [36] (as shown in Figs. 5 and 6, respectively). In Fig. 5, we analyze the regions that MSUN is concerned with for each category via the heat map generated using Grad-CAM, and whether the network learns the correct features by the areas that the network is focused on. The figure shows that the extracted feature representation using MSUN determines the location of plant leaf diseases more accurately than using DANN. In Fig. 6, we exploit t-SNE to visualize the adaptation performance during the transfer (PVD→C-PD) using MSUN and other state-of-the-art methods. The figure shows that the baseline method that directly transfers from the source domain to the target domain without domain adaptation fails to achieve an effective performance due to the domain shift, where both feature distributions of the 2 domains and intradomain class features are not well distinguished. In addition, although existing methods perform better than the baseline, they still suffer from the ambiguous boundary between different feature classes and scattered distribution within the same feature class, which easily lead to inaccurate classification. This is because these methods fail to address the problem existing in our task that the images from the laboratory and field have a more extensive domain divergence compared to the general domain adaption task. In contrast, by aligning the subdomains, i.e., the embedded features belonging to each class, the proposed MSUN is capable of not only aligning the features of each class between source and target domains but also distinguishing the features of different classes from the target domain. Therefore, we conclude that MSUN is rather effective for alignment when dealing with the large domain shift occurring in our plant disease task.

## Conclusion

In this paper, a novel multirepresentation subdomain adaptation network with uncertainty regularization (MSUN) is proposed for plant disease recognition in the field environment. The proposed MSUN effectively addresses the uncertainty of various diseases across species (i.e., large interdomain discrepancy, large intraclass discrepancy within domains, and fuzzy boundaries between classes within domains) that existing UDA methods failed to focus on. We incorporate multiple representations with subdomain alignment, allowing the network to learn more global structural information while concentrating fine-grained information and achieving semantic alignment at the class level. To suppress the uncertainty in the transfer process (i.e., the accumulation of pseudo-labeling errors), we also introduce auxiliary regularization, which enables our model to be more effective. Our work provides insight into the problem of plant disease recognition. In addition, our model allows for a feed-forward model alone, without the need for adversarial training, and is back-propagated to train the network efficiently. Note that our model yields striking results on several challenging datasets.

## Data Availability

All the used datasets are publicly available.
